# Knockout mice reveal a role for protein tyrosine phosphatase H1 in cognition

**DOI:** 10.1186/1744-9081-4-36

**Published:** 2008-08-12

**Authors:** Claudia Patrignani, Maria Chiara Magnone, Patrizia Tavano, Michele Ardizzone, Valeria Muzio, Béatrice Gréco, Paola F Zaratin

**Affiliations:** 1 MerckSerono Ivrea, Colleretto G. (TO) 10010, Italy; 2 University of Eastern Piedmont, Novara, Italy

## Abstract

**Background:**

The present study has investigated the protein tyrosine phosphatase H1 (PTPH1) expression pattern in mouse brain and its impact on CNS functions.

**Methods:**

We have previously described a PTPH1-KO mouse, generated by replacing the PTP catalytic and the PDZ domain with a LacZ neomycin cassette. PTPH1 expression pattern was evaluated by LacZ staining in the brain and PTPH1-KO and WT mice (n = 10 per gender per genotype) were also behaviorally tested for CNS functions.

**Results:**

In CNS, PTPH1 is expressed during development and in adulthood and mainly localized in hippocampus, thalamus, cortex and cerebellum neurons. The behavioral tests performed on the PTPH1-KO mice showed an impact on working memory in male mice and an impaired learning performance at rotarod in females.

**Conclusion:**

These results demonstrate for the first time a neuronal expression of PTPH1 and its functionality at the level of cognition.

## Background

Tyrosine phosphorylation plays an important role in several signaling pathways regulating cell growth, differentiation, cell cycle, apoptosis and neuronal functions [1,2]. The phosphorylation/dephosphorylation balance is controlled by protein tyrosine kinases and phosphatases. PTPs can be distinguished into four classes: 1) classical PTPs that can be subdivided into transmembrane, receptor-like enzymes, and the intracellular, nonreceptor PTPs, 2) dual-specificity PTPs (Ser and Tyr phosphatases), 3) low molecular weight PTP and 4) the Asp-based PTPs (Tyr/Ser phosphatase activity) [3].

Classical PTPs have been reported to play a key role in neural functions, from development to cognitive function. For example, RPTPs such as PTPδ, PTPσ, LAR, and especially PTPRO, are important players in axonal growth and guidance during development [4]. Studies on PTPσ-KO (RPTP) mice have shown involvement of this PTP in the regulation of the developing hypothalamo-pituitary axis [5,6] and in the development of the CNS architecture [7]. PTPBL-KO (non receptor like PTP-NRPTP) mice display impaired motor nerve repair in a model of sciatic nerve crush lesion [8] and PTPMEG (NRPTP) interacts with key intracellular players leading to the stimulation of the channel activity of NMDA receptors [9].

In the present study we focused our attention on a NRPTP, PTPH1, and on its possible role on neural functions. Indeed PTPH1 has been shown to be expressed in the CNS [10] but little is currently known on its potential impact on CNS functions. PTPH1 (also called PTPN3) belongs to a sub-family of non receptor cytosolic PTPs characterized by the presence of a FERM domain (band 4.1, ezrin, radixin, moesin) at its N-terminus, responsible for the interaction with transmembrane proteins and/or phospholipids in the cell membrane [11-13]. In addition PTPH1 has a PDZ domain in the central part responsible for the interaction with other proteins, whereas the single catalytic domain is located at the C-terminus.

PTPH1 activity has been involved in a variety of cellular functions including TCR-signaling [14-16], cell cycle regulation [11,16,17], endoplasmic reticulum assembly [18], cardiac sodium channel modulation [19] and TNFα converting enzyme inhibition [20].

Recently, our group has demonstrated that PTPH1 dephosphorylates GHR *in vitro *and in cellular assays [21] and results in an increase of body weight in the functional PTPH1-knockout (KO) mice via modulation of IGF1 secretion [22] thus demonstrating its *in vivo *relevance.

PTPH1 has been shown in the rat to be highly expressed in thalamic nuclei as well as various cortical areas [10]. However, no information is currently available on its impact on CNS functions. To address this question we have further characterized our PTPH1-KO mice line through behavioral and anatomical approaches. PTPH1 expression and localization was evaluated by LacZ staining in the brain and a behavioral test battery evaluated PTPH1 loss on CNS functions such as locomotor activity (open field), anxiety-like behavior (open field and elevated plus maze), motor ability, coordination and learning (accelerating rotarod), spatial working memory (Y maze) and nociceptive sensitivity (hot plate).

## Methods

### Animals

PTPH1-KO and wild type littermates (F2 generation, 87.5% C57Bl/6 – 12.5% 129S6SvEv) aged 3–4 months were used for behavioral phenotyping. Mice were individually caged and maintained in a 12:12 hours light: dark cycle (lights on at 7 am) at 21 ± 1°C with food and water available *ad libitum*. Protection of animals used in the experiment was in accordance with Directive 86/609/EEC, enforced by the Italian D.L. No. 116 of January 27, 1992. Physical facilities and equipment for accommodation and care of animals were in accordance with the provisions of EEC Council Directive 86/609. Tail snips from mice were collected and genotyped as previously reported [22].

### PTPH1 KO design

PTPH1-KO mice were generated using the Velocigene technology [23], as described in details elsewhere [22]. Briefly a mouse BAC containing the PTPH1 gene was modified: an in-frame LacZ reporter sequence and a neomycin-selectable marker replaced exons 15 to 22 encoding for the PDZ and the catalytic domain of PTPH1. BAC electroporation into embryonic stem cells was performed. F1 heterozygous mice were bred to generate F2 PTPH1-KO mice. Line breeding and animal care were performed in Charles River Italy and France.

### LacZ staining procedure and immunohistochemistry

PTPH1-KO and WT mice, 12 months old, n = 2, male and females, were sacrificed by ip overdose of thiopental (5%), perfused with paraformaldehyde 4%, then washed in PBS and incubated overnight at 37°C in the solution containing the substrate for beta-galactosidase (beta-gal, encoded by the LacZ cassette) coupled to a NBT salt. The organs and the tissues in the sections display a green/blue staining where PTPH1 gene is normally expressed. After rinsing into PBS, organs were postfixed in PFA 4% for 1 hour, then incubated in 50% glycerol overnight at 4°C and finally maintained in 70% glycerol at room temperature. LacZ staining was observed through a low magnification microscope and described by an operator blind to the genotypes.

LacZ staining was also performed on CNS sections. Mice (n = 3, 12 months old) were sacrificed by ip injection of an overdose of thiopental (5%), perfused with PBS and PFA 4%. Brains were removed and postfixed overnight at 4°C in PFA 4%, then placed overnight at 4°C in 15% and finally in 30% sucrose buffer. The brains were then included in O.C.T. (Tissue-Tek) and sections were cut on slides with a cryostat at 20 μm thickness. The slides were incubated in LacZ staining solution (see above) overnight at 37°C, washed thrice in PBS (5 min each) and either counterstained with H&E (Merck KGaA) [23] or co-expressed with NeuN immunostaining. Briefly, sections were incubated for 3 hours at room temperature in blocking solution (Vectastain Kit), washed in TBS (Tris-buffered saline), incubated overnight at 4°C with a solution containing the primary antibody mouse anti-mouse Neuronal Nuclei (Chemicon MAB377, 1/1000). The staining was revealed by ABC kit secondary antibody (mouse Vectastain Kit), and DAB (Sigma). After dehydration, sections were transferred onto coverslips. LacZ staining and co-expression with NeuN-immunoreactivity (NeuN-ir) was observed by microscopy and described by an operator blind to the genotypes.

### Semiquantitative RT-PCR for beta galactosidase gene

Semiquantitative RT-PCR for PTPH1 and beta-gal gene expression was performed in different brain areas of PTPH1-KO and WT mice in order to confirm the presence of beta-gal expression in the KO tissues, replacing PTPH1 PDZ and catalytic domain. Brains from KO and WT mice (n = 5, 6 months old) were freshly removed and rinsed in HBSS. Hippocampus, cerebellum, cortex, striatum, midbrain and olfactory bulbs were dissected. Total RNA was extracted using Trizol Reagent (Invitrogen) and cleaned-up by RNAeasy columns from Qiagen. 5 μg of total RNA were used to perform the RT-PCR reaction (SuperScript II RT kit, Invitrogen). The primer sequences for LacZ amplification were the following: LacZ – forward 5'-GAT GTA CGT GCC CTG GAA CT/reverse 5'-GGT CCC ACA CTT CAG CAT TT. In order to load equally the reaction mixes, a 300 bp fragment of Histone 2A was amplified as a house keeping gene with the following primers: H2Az forward – 5' CGT ATT CAT CGA CAC CTG AAA; H2Az reverse – 5' CTG TTG TCC TTT CTT CCC GAT.

### Behavioral phenotyping test battery

Neurological functions of PTPH1-WT and KO mice (males and females, 11 weeks-old, n = 10 per gender per genotype) were assessed through a behavioral test battery.

The sequence of the test battery was chosen from the least invasive to the most ones. The schedule of the testing sessions included one week of recovery from one test to the next, as reported in Table 1.

**Table 1:** Schedule of the behavioral test battery.

**Age (wks)**	8	9	10	11	12	13	14	15
**10M+10F**	arrival	quarantine	adaptation	**Open Field**	**EPM**	**Rotarod**	**Y-maze**	**Hot plate**

### Open field

After one hour of adaptation in the testing room, each mouse was placed in an open field chamber (50 cm^2 ^wide with white floor and walls) (ViewPoint Life Sci. Inc.) to test locomotor activity and anxiety-like behaviors. Locomotion was recorded for one hour by a video camera and analyzed automatically by VideoTRACK^® ^software (ViewPoint Life Sci. Inc.). Locomotor activity was evaluated by calculating the total path length traveled, whereas the relative time spent in the center was taken as indicative of anxiety-like behavior [24]. The tests were performed in two sessions with equivalent group representation.

### Elevated plus maze

After one hour of adaptation in the testing room, anxiety-like behavior was tested for each mouse by EPM within one session. The apparatus consists of four arms (29.5 cm long and 5 cm wide each). Two arms are open whereas the 2 others are limited by 2 black walls (20 cm high). The number of entries of each mouse in the open and closed arms was recorded by a video camera during a period of 5 minutes and analyzed by the SMART Video-Tracking Software (ViewPoint Life Sci. Inc.). The total number of entries into the arms is an index of locomotion, whereas the percentage of time spent and percentage of entries in the closed arms is an index of anxiety-like behaviors [25].

### Accelerated rotarod

Motor ability, coordination and learning were evaluated by using an Accelerated Rotarod apparatus for mice (Cat. # 7650 by Jones and Roberts, distr. by Basile Instr., Italy). The apparatus was placed within the animal colony room and was cleaned after each trial. Mice were tested for their abilities to maintain a balance on a rotating bar, which accelerated from 4 to 40 rpm/min in a 5 min trial. Latency to fall off was measured within one session and all mice underwent four trials (one every 30 min) [26-28]. The differences at the rotarod performances in WT and KO were assessed by a single set of trials [27,28]. This set-up allows a major focus on the early phases of motor learning, involving a strong activation of prefrontal cortex and of the associative areas of basal ganglia and cerebellum [29,30].

### Y-maze alternation

After one hour of adaptation in the testing room, mice were tested on a Y maze apparatus (40 cm long/8 cm wide arms with transparent walls) to investigate spatial working memory [31]. The number and the sequence of the arm entries for each mouse were recorded during 5 minutes. The locomotion index was calculated as the overall number of arm entries, whereas the working memory index was calculated as following: number of exact alternations (entries into three different arms consecutively)/possible alternations (i.e. the number of arms entered minus 2) × 100.

### Hot plate

Thermal sensitivity was assessed by a hot plate apparatus for mice (Cat. # 7280 by Biol. Research Apparatus, distr. by Basile Instr., Italy) and lasted a maximum of 45 seconds, time at which damages could occur [32]. The apparatus was placed in the animal colony room and all the mice were tested within one session. Animals were placed on a surface heated at 52.5°C and the latency (seconds) to shake or lick the paw was recorded by the operator.

### Statistics

Statistical comparisons were performed by unpaired two-tailed T-test (p < 0.05) and two-way ANOVA (p < 0.05) followed by post-hoc test as necessary. In the accelerated rotarod, two-way ANOVA with repeated measures followed by T-test was used. Results are expressed as mean ± SEM.

## Results

### LacZ staining in whole mount

**Figure 1:**
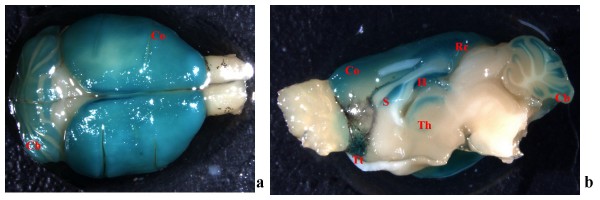
PTPH1-KO adult mouse brain. ***a***: whole brain, dorsal view, staining in cerebellum (Cb) and cortex (Co); ***b***: LacZ staining on brain, sagittal view: detection in the tenia tecta (Tt), cortex (Co), thalamus (Th), hippocampus (H), retrosplenial cortex (Rc), septum (S) and in the granule cell layer of cerebellum.

In PTPH1-KO adult animals, LacZ staining in the brain was observed in the cerebellum, hippocampus and in the thalamic nuclei. In addition a strong staining was observed in the cerebral cortex, tenia tecta and septum (Figures 1a, 1b).

### LacZ staining in sections and RT-PCR results

No gross cytoarchitectural brain differences were observed by simple visual observation at the microscope in the cortex, hippocampus and thalamus in PTPH1-KO mice compared to WT littermates.

LacZ staining was performed on frozen brain sections to confirm and to describe the expression of PTPH1 at the brain structural level (Table 2).

**Table 2:** Qualitative estimation of LacZ staining intensity in the different brain areas.

**Brain Area**	**Intensity of LacZ staining**	**Brain Area**	**Intensity of LacZ staining**
Cerebral cortex	+	Dorsal Tenia Tecta	++
Retrosplenial cortex	++	Septohippocampal nu	+
CA1 oriens layer	+++	VPL	+
CA1 radiatum layer	+++	MDL	++
CA1 pyramidal cell layer	+	AV	++
CA2 oriens layer	-	VPM	++
CA2 radiatum layer	-	VL	+
CA2 pyramidal layer	+	VM	++
CA3 oriens layer	+	Po	++
CA3 radiatum layer	+/-	LD	+
CA3 pyramidal layer	+	Rt	+
DG granular cell layer	-	DLG	++
DG molecular layer	+++	VPPC	++
DG hilus	-	PF	-
Fascicola cinereum	++	cerebellum	+
Indisium griseum	+		

-: absent; +/-: faint; +: present; ++: intense; +++: very intense staining. Cornu Ammonis (CA), Dentate Gyrus (DG), mediodorsal lateral (MDL), anteroventral (AV), ventromedial (VM) ventral posteromedial (VPM), ventrolateral (VL), ventral posterolateral (VPL), laterodorsal (LD), posterior (Po) and reticular (Rt), ventral posteromedial parvicel (VPPC) thalamic nuclei, lateral geniculate nucleus (DLG), parafascicular thalamic nuclei (PF), nuclei (nu).

**Figure 2:**
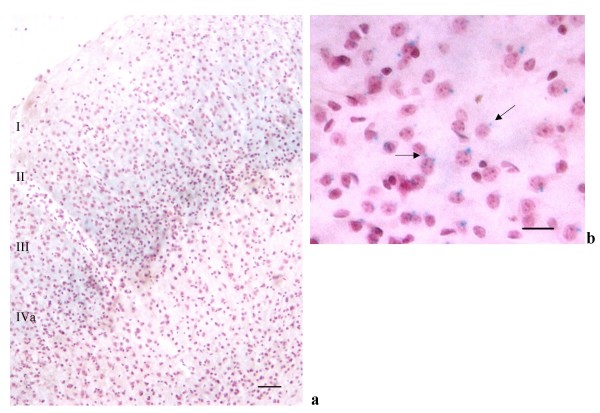
***a***: PTPH1-KO cerebral cortex (10×, scale bar: 220 μm). ***b***: positive cytoplasmatic and perinuclear LacZ staining (blue dots) in the external pyramidal (III) and internal granular layer (IVA) (63×, scale bar: 10 μm).

**Figure 3:**
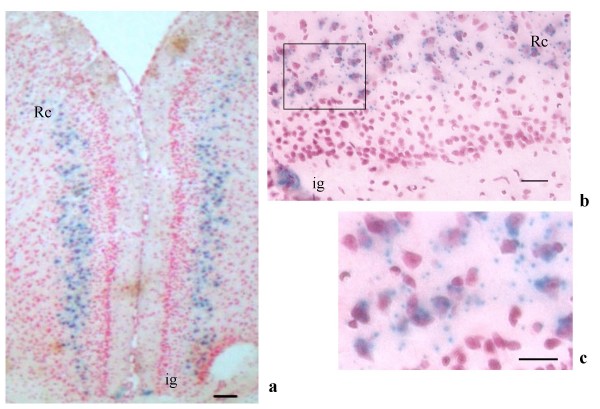
PTPH1-KO cerebral cortex. ***a***: LacZ detection in retrosplenial cortex (Rc) and indusium griseum (ig) staining (4×, scale bar: 80 μm). ***b***: detail of the Rc and ig (40×, scale bar: 20 μm); ***c***: positive cytoplasmatic staining of the neurons of Rc (63× scale bar: 10 μm); the interneural LacZ signals are due to the presence of trans-sectioned axons and dendrites.

In cortical regions, LacZ was expressed in the external pyramidal (III) and internal granular layer (IVA) of the cerebral cortex (Figures 2a, 2b), in the retrosplenial cortex (Figures. 3a, 3b 3c, 4a and 4b) and indusium griseum (Figures 3a, 3b). In the cerebellum, in spite of a strong staining in the whole mount (Figure 2), only a faint LacZ signal was observed in sections (Figures 5a, 5b) in particular in the granule cells, close to the nuclei. The RT-PCR on cortical and cerebellar extracts confirmed the presence of LacZ expression in these brain areas (Figure 6).

**Figure 4:**
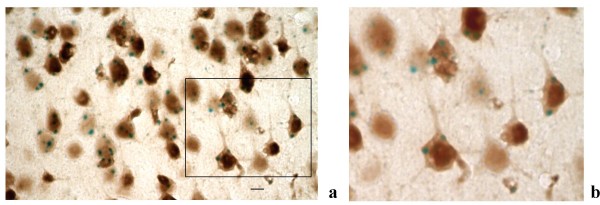
PTPH1-KO cerebral cortex. ***a***: colocalization of NeuN-ir and LacZ staining signal in the Rc (100×, scale bar: 4.5 μm).; ***b***: detail of the cytoplasmatic signal of LacZ in neurons.

**Figure 5:**
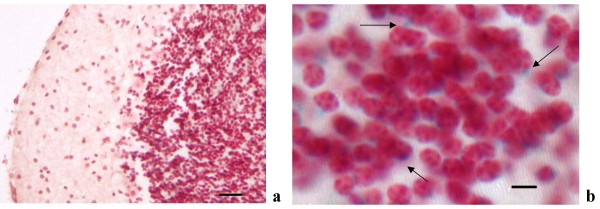
PTPH1-KO cerebellar cortex. ***a***: faint LacZ staining in the granule cell layer (20×, scale bar: 16.5 μm); ***b***: perinuclear staining in the granule cell layer of the cerebellum (63×; scale bar: 5 μm).

**Figure 6:**
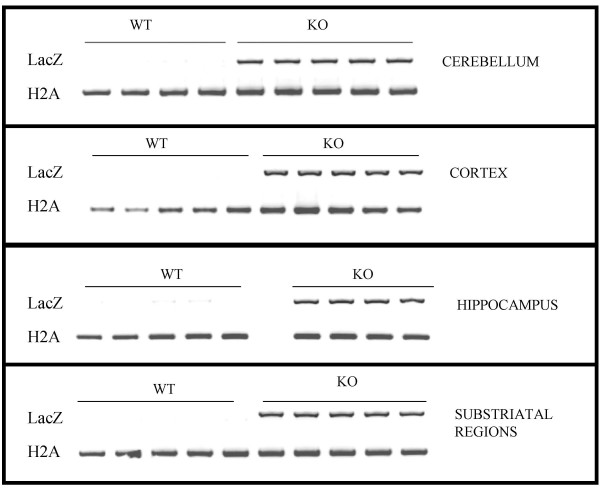
RT-PCR for beta-galactosidase expression in brain extracts. Beta-gal mRNA is expressed in PTPH1-KO cerebellum, cortex, hippocampus and substriatal regions (midbrain, thalamic nuclei, pontine region); no beta-gal expression detected in WT brain extracts (first lane of each block); histone H2A gene was used as positive control (second lane).

**Figure 7:**
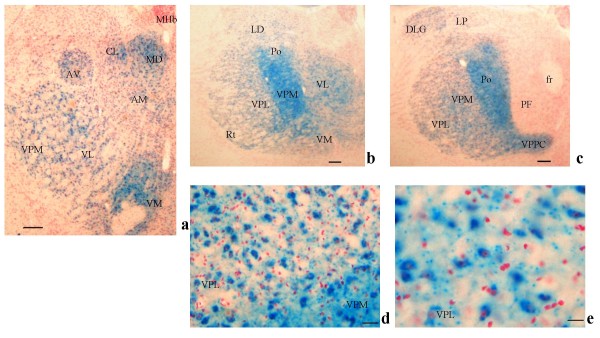
PTPH1-KO thalamus [76]. ***a***: LacZ expression detected in several thalamic nuclei (4×; scale bar: 165 μm): mediodorsal (MD), central lateral (CL), anteroventral (AV), anteromedial (AM), ventromedial (VM) ventral posteromedial (VPM) and ventrolateral (VL) thalamic nuclear groups. MHb: medial habendular nuclei. ***b***: LacZ expression detected in the ventral posteromedial thalamic nuclei (VPM) and it is present also in ventrolateral (VL), ventromedial (VM), ventral posterolateral (VPL), laterodorsal (LD), posterior (Po) and reticular (Rt) thalamic nuclei (2.5×, scale bar: 130 μm). ***c***: LacZ is expressed in the dorsal lateral geniculate nucleus (DLG) and in the lateroposteral thalamic nuclear group. In this caudal section LacZ staining is more intense in the posterior nucleus, but present also in VPM, VPL and VPPC (ventral posteromedial parvicel) thalamic nuclei (2.5×, scale bar: 13 μm). ***d***: Detail of beta-gal expression in neural cell body of VPL and VPM at 40× (scale bar: 20 μm) and ***e***: at 63× (scale bar: 10 μm).

**Figure 8:**
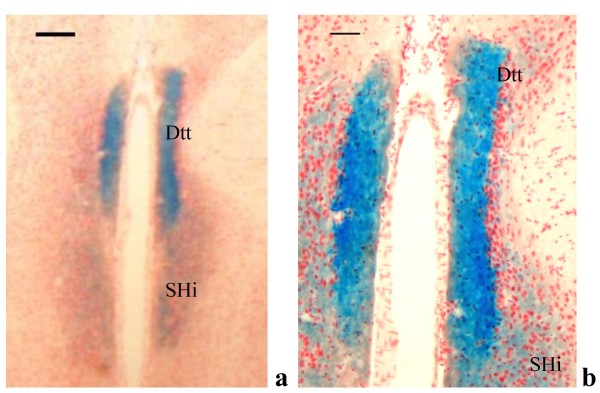
PTPH1-KO adult mouse brain. ***a***: beta-gal expression detected in the dorsal tenia tecta (Dtt) and in the septohippocampal nuclei (SHi) (4×; scale bar: 165 μm). ***b***: Detail of cytoplasmatic LacZ staining in the Dtt and SHi (10×; scale bar: 70 μm).

In subcortical regions, LacZ was detected in the anterior ventral, mediodorsal, ventrolateral, anteromedial and central lateral thalamic nuclear groups (Figure 7a). In more caudal thalamic areas, LacZ was again detectable in the posterior thalamic nuclear group (Po), and to a lesser extent in posteromedial, in posterolateral and in reticular thalamic nuclei and also in the dorsal lateral geniculate nuclei (Figures 7b, 7c, 7d and 7e). In the tenia tecta, LacZ staining visible in the whole mount preparation was confirmed (Figures 2, 8a, 8b). The RT-PCR on substriatal regions including the thalamus, the midbrain and the pontine areas confirmed the presence of LacZ expression in some of these brain areas (Figure 6). To exclude any potential impact of LacZ blood signal contamination in brain areas, RT-PCR on 5 to 20 μl of whole blood was carried out and did not reveal any significant signal [Additional files 2, 3].

**Figure 9:**
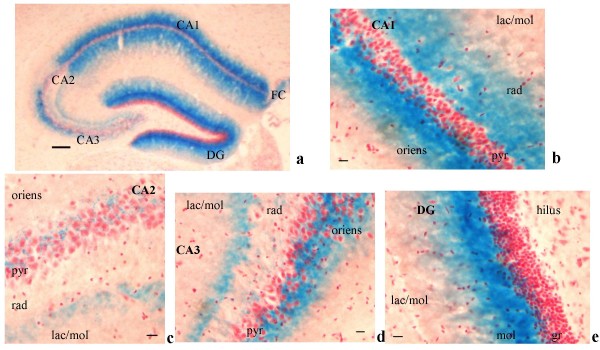
PTPH1-KO hippocampus [76]. ***a***: Hippocampus at 4×; ***b***: CA1 area of hippocampus shows very intense LacZ staining in both oriens and radiatum layers and to a less extent in the pyramidal cell layer (20×; scale bar: 90 μm). ***c***: CA2 area of hippocampus displays LacZ-positive staining in the pyramidal cell layer. ***d***: CA3 area shows an intense beta-gal expression in the oriens and pyramidal cell layer, and in a less extent in the radiatum (20×). ***e***: The dentate gyrus (DG) displays a strong LacZ staining in the molecular layer and not in the hilus (20×) (scale bar: 20 μm). pyr: pyramidal cell layer; oriens: oriens layer; rad: radiatum layer; mol: molecular layer: gr: granule cell layer; lac/mol: lacunosum-molecular layer.

In the hippocampus, LacZ expression was observed in the cytoplasm of a few pyramidal cells and through the fibers of the oriens and radiatum layer in a rostral caudal spread (Figure 9a). In rostral sections, LacZ was expressed in the septohippocampal nuclei (Figure 8a). In more caudal sections LacZ was present in the CA1 and CA3, and in a lesser extent in the CA2 (Figures 9b, 9c and 9d). In the CA3 LacZ was strongly expressed in the oriens and pyramidal cell layer (Figure 9d), but its intensity was reduced in the radiatum and oriens compared to CA1 (Figure 9b). No staining was detected in the lacunosum-molecular layer in CA1, CA2 and CA3 (Figures 9b, 9c and 9d). The dentate gyrus showed a strong positive LacZ signal in the molecular layer, but not in the hilus (Figure 9e).

### Behavioral phenotyping

As previously demonstrated, PTPH1-KO mice were healthy, reproduced normally and did not show any phenotypic traits distinguishing them from their WT littermates by simple visual observations [22,33]. An increased body weight has been detected in PTPH1-KO mice compared to WT littermates, more pronounced in male mice and probably due to an enhanced GHR sensitivity, that leads to increased IGF-1 mRNA and protein expression in liver and plasma, respectively [22].

In EPM, open field test and hot plate tests (anxiety-related behavior and thermal pain sensitivity), PTPH1-KO male and female mice did not show any significant differences in comparison with their WT littermates (data not shown).

**Figure 10:**
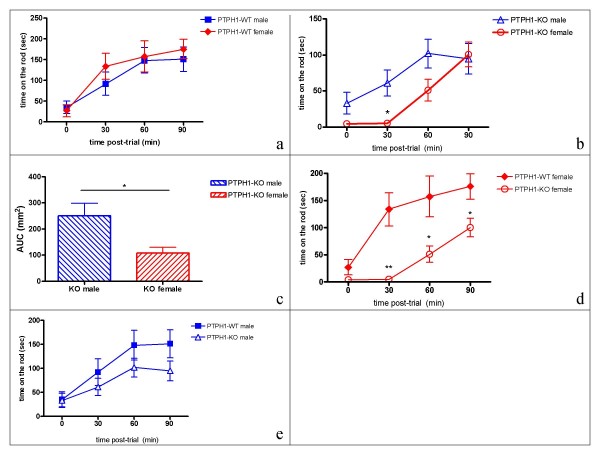
Rotarod test on PTPH1-WT and KO mice (n = 10) males and females. ***a***: WT males and WT females do not display any significant different performance at the rod (P_2WAY _= 0.5824) ***b***: KO males and KO females display a significant different performance (P_2WAY _= 0.007) (post-hoc T-test: P_0 _= 0.109; P_30 _= 0.015; P_60 _= 0.067; P_90 _= 0.835). ***c***: 50% difference in the area under the curve represented in figure 10a (unpaired T-test, P=0.0194). ***d***: Female KO mice display a worse performance at the rod compared to WT females (P_0 _= 0.171; P_30 _= 0.002; P_60 _= 0.028; P_90 _= 0.025) ***e***: No significant difference in the performance on the rod between male KO and WT mice. ***a, b, d, e***: All the data were analyzed by Two-way Anova followed by T-test; *: p < 0.05; **: p < 0.01.

In the accelerated rotarod and Y-maze test, significant differences were observed between PTPH1-KOs and WTs based on gender and genotype factors. In the accelerating rotarod test PTPH1-WT mice did not show any gender differences (P_2WAY _= 0.5824 (WT gender vs WT activity); P_AUC _= 0.3218 (PTPH1-WT male vs female)) (Figure 10a). PTPH1-KO male mice displayed an overall significant better performance compared to their matched female littermates (P_2WAY _= 0.007 (KO gender vs KO activity) (Figure 10b). Post-hoc T-test analyses showed that the difference was significant at the second trial of the test (P_0 _= 0.109; P_30 _= 0.015; P_60 _= 0.067; P_90 _= 0.835), and the area under the curve for PTPH1-KO male mice was significantly higher (by 50%) compared to the matched values of the female littermates (P = 0.0194) (Figure 10c).

Considering this gender effect, the follow up analysis was carried out in males or females assessing genotype effects on activity. PTPH1-KO female mice performed significantly worse compared to their matched WT littermates, starting from the second trial and onwards (P_0 _= 0.171; P_30 _= 0.002; P_60 _= 0.028; P_90 _= 0.025) (Figure 10d). No significant differences were observed in PTPH1-KO male mice compared to their matched WT littermates (P_0 _= 0.92; P_30 _= 0.363; P_60 _= 0.222; P_90 _= 0.135) (Figure 10e).

**Figure 11:**
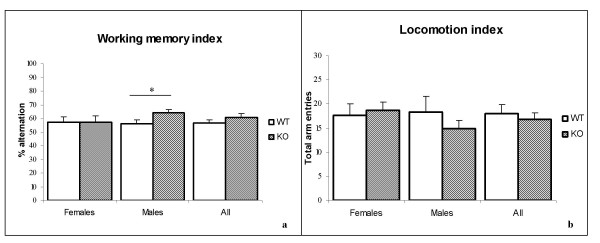
Y-maze behavioral test on PTPH1-WT and KO mice (n = 10) males and females. ***a***: Male KO mice display higher working memory index compared to WT male littermates (P_male _= 0.041); no differences recorded in the female mice. ***b***: No significant differences recorded in the locomotion index, represented by the total arm entries between PTPH1-WT and KO males and females. T-test, *: p < 0.05.

In the Y-maze test, no differences were detected between PTPH1-KO and WT female mice either in working memory (P_female _= 0.972) or in locomotion indices (P_female _= 0.73; Figures 11a, 11b). On the other hand, PTPH1 KO male mice displayed a significantly higher working memory index (percentage of exact alteration; P_male _= 0.041) but similar locomotion activity (total arm entries) (P_male _= 0.348) compared to their matched WT littermates (Figures 11a, 11b).

## Discussion

PTPs are key factors in multiple signaling pathways, leading to modulated functional activities in various cell types [34,35]. Among all PTP forms, PTPH1 has been shown *in vitro *to modulate cardiac sodium channel Na_v_1.5 [19], that it is also known to be expressed in the axons of cerebral cortex, cerebellum, thalamus and brain stem [36]. Moreover, PTPH1 contains a domain with high sequence homology with the members of the band 4.1 superfamily protein, FERM. This domain mediates the linkage of actin filaments to the plasma membrane [37], and therefore may be involved in cytoskeleton-membrane interactions, crucial for axon functionality. To further understand the potential role of PTPH1 in neural functions *in vivo*, we first investigated its expression pattern in embryonic and adult PTPH1-KO mice CNS by LacZ staining, and second its role in CNS functions by behavioral phenotype characterization.

In rat embryonic stage Es19, PTPH1 expression through FISH analyses has already been shown in the dorsal thalamic nuclei, which give rise to the thalamo-cortical connections in adulthood [10]. Thus, it has been suggested to play a role in the maintenance of these connections in adults. We replicated these data in PTPH1-KO mice at Es14 and Es16 embryonic stages. PTPH1 is expressed in the hypothalamic area and but also in the dorsal root ganglia of the spinal cord, excluding the spinal cord itself [Additional file 1] [38]. Moreover, at postnatal P1, PTPH1 expression is also present in peripheral organs such as muscles and intestines as in the adults [22]. On the other hand, the CNS expression at P1 appears weaker than in the adults suggesting a pattern of PTPH1 expression corresponding to specific developmental stages of the CNS as well as peripheral organs (data not shown). These changes in expression may play a role in various developmental functions that need to be further understood.

In PTPH1-KO adults, LacZ is expressed in different CNS areas such as cerebral and retrosplenial cortices (Figures 1, 2, 3 and 4), hippocampus (Figure 9), thalamus (preferentially ventral thalamus) (Figure 7), cerebellum (Figure 5) and in the region of the tenia tecta (Figures 1, 8). This data confirms previously observed expression patterns in the rat brain by Sahin et al. [10] and extends the observation to other brain regions. We, furthermore, demonstrate that PTPH1 is expressed within the cytoplasm and close to the cell membrane of neurons in most of the brain area investigated (Figures 4a, 4b). It is known that the FERM domain is indeed necessary for PTPH1 localization close to the plasma membrane in Jurkat T cells [14] and it could be responsible for the punctate expression pattern of PTPH1 in the cytosol of the neurons (Figure 4b) [39]. This supports the concept that PTPH1 may be involved in cytoskeleton-membrane interaction within extended neuronal population in the CNS, potentially playing a role in various neuronal functions.

Indeed the neural expression of PTPH1 in CA1, CA3 and DG of the hippocampus (Figures 9a, 9b, 9c, 9d and 9e), in the retrosplenial cortex (Figures 3, 4) and in a series of thalamic nuclei (Figures 7a, 7b, 7c, 7d and 7e) suggests an involvement of PTPH1 in the modulation of the memory circuit. Both hippocampus and retrosplenial cortex are key regions in the spatial working memory functions [40-46]. Moreover, several thalamic nuclei have also been shown to be important in the memory process [47,48]. For example, a strong loss of dorsomedial and ventral posterior thalamic neurons is associated with severe cognitive and memory disabilities in patients affected by traumatic brain injury [49]. Lesions in the lateral thalamus may lead to important working memory defects in rodents [50]. The anterior thalamic nuclei project via the retrosplenial cortices to the hippocampus [51,52], thus underlying the importance of both these circuits and of PTPH1 in the mnemonic process.

Another interesting PTPH1-positive area is the indusium griseum (Figures 3a, 3b) whose role in the adult brain is not clear. It is thought to be part of the limbic system, receiving afferents from the entorhinal and pyriform cortex and projecting to the septohippocampal nuclei, olfactory tubercle (presumably the tenia tecta) and the medial frontal cortex [53,54]. The expression of PTPH1 in these specific regions suggests a potential role in the processing/integration of memory and sensory information to the SHi and likely the cortex.

Indeed PTPH1 expression is also detectable in the pyramidal neurons in layer III and IVA of the cerebral cortex of the mouse (Figures 2a, 2b), in agreement with Sahin's findings in the rat brain. The middle layers (III and IV) of the cerebral cortex are key sites for thalamic inputs [55,56] especially for VPM and VPL, primary thalamic nuclei for somato-sensory information integration [57]. Furthermore a strong cortico-cortical communication has been assessed between these two layers [58], thus suggesting a role for PTPH1 as key regulator in the transmission of the thalamo-cortical and cortico-cortical information.

The cerebellar cortex is also positive for PTPH1 expression, in particular in the cytoplasm of granule cells (Figure 5b). The cerebellum is known to be the main structure for motor learning functions. In particular, the cerebellar cortex seems to be involved in the early learning phases of motor activities [59,60] that include also a strong activation of other areas such as prefrontal cortex and basal ganglia [29,30]. PTPH1 expression in the granule cells seems to indicate a potential involvement in the processing of afferent information to the purkinje cells, since it is known that afferents fibers to the cerebellar cortex will project in part through the granule cell layer.

PTPH1 expression pattern observed in our analysis points out a potential involvement of this phosphatase in numerous CNS processing functions such as locomotion, sensorial integration, learning and memory. In this study, the behavioral phenotyping of the PTPH1-KO mice allowed us to test these hypotheses *in vivo*. Indeed, as already demonstrated by our group [22] and also by others [33], PTPH1-KO mice are healthy and do not display any phenotype, distinguishing them from their matched WT littermates, detectable by simple visual observation. Therefore PTPH1-WT and KO mice underwent a battery composed by five behavioral tests, from the least to the most invasive (Table 1), with the tolerable limitation of the handling bias.

Behavioral testing revealing locomotor dysfunctions, such as open-field, EPM and Y-maze did not highlight differences between the two genotypes (Figure 11b), suggesting that PTPH1 does not play a critical role in the integration of locomotor information.

Anxiety-like behaviors measured by open-field (as path in the center) and EPM (as time spent in the open arms), exploiting rodents natural aversion to open space, did not show any differences between the two genotypes (data not shown), leading to the conclusion that PTPH1 may not be involved in the integration of thalamo-limbic information, key paths for anxiety behavior processing. Similar conclusions can be drawn from the lack of difference between the genotypes regarding integration of nociceptive information, based on hot plate test.

In the behavioral test, that partly depends on working memory performances (Y maze), PTPH1-KO male mice showed a slightly better short-term memory than their WT littermates (Figure 11a). Thus, PTPH1 may be involved in the integration of memory information. This was further strengthened by results obtained with a test assessing learning and coordination, the rotarod. Contrary to other behaviors where little differences have been observed, learning and coordination capacities in PTPH1-KO female mice are significantly impaired (Figures 10b, 10c). The low rotarod performance on the early trials, compensated by the last trial, is suggestive of a delay in learning acquisition (Figures 10b, 10d).

As reported in Pilecka et al., our PTPH1-KO mice express the non-catalytic part of PTPH1 in frame with the enzymatically active part of LacZ gene. LacZ is widely used as a reporter for promoter activity in KO mice and all those mice express a modified protein, whose full function is not known. So far it was never reported a function of LacZ alone in cognition and we consider quite unlikely that this is the case in our mice. Thus, it is very likely that the behavioral phenotype we detect in our mice is linked to the deletion of the catalytic domain of PTPH1.

The impairment in learning and coordination of PTPH1-KO female mice may be resulting from the involvement of PTPH1 in the GH signaling pathway [21]. Indeed our group has already shown that PTPH1-KO mice display higher GHR response *in vivo *and consequently a higher expression of its down-stream effector hormone, the IGF1 in liver and plasma [22]. GHR is highly expressed in most areas of the CNS, in particular in the choroid plexus, hippocampus, putamen, thalamus and hypothalamus. Similarly IGF1 and IGF1-receptors are localized predominantly in hippocampus, but also in amygdala, cerebellum and cortex [61]. Although IGF1 is considered a neuroprotective hormone, it can be produced in the CNS, it is primarily synthesized in the liver and can cross the blood-brain barrier [62-65]. The GH-IGF1 axis is also known to influence cognitive functions due to several neuroprotective effects on the hippocampus [66]. Furthermore it has been recently pointed out that old conditional liver-IGF1-KO mice display impaired spatial learning and memory [67]. The presence of PTPH1 in key CNS regions, as well as the consequent deregulation of the GH-IGF1 axis in KO mice, strengthens the concept that the PTPH1 network (CNS and downstream peripheral effectors) may be involved in cognitive functions.

The behavioral tests assessing working memory and specifically learning revealed not only a genotype effect but also a gender effect, as mentioned above. Sex hormones are known to modulate the somatotropic system [68,69]. In humans, testosterone has an important effect on GH axis, in part by its aromatization to estradiol. Administration of estrogens, or aromatized androgen, modulates GH axis neuroregulation [69,70]. In particular, chronic E2 administration has been shown to reduce GH-induced IGF1 increased expression in liver and plasma via a negative feedback mechanism, while acute E2 administration leads to the expected GH-induced IGF1 release [71]. Furthermore, it has been reported that estrogens play not only regulatory functions on neuroendocrine systems but can also have stimulatory or inhibitory impacts on the inter-connectivity of the hippocampal structure depending on the gender [72-75], meaning that the same stimulus can have opposite effects in male *vs *female mice. Thus, the cognitive behavioral differences observed in our KO mice are underlying the potential impact of the PTPH1 network on neuroendocrine regulation as well as on cellular architecture within specific brain regions.

## Conclusion

In conclusion, we have demonstrated that PTPH1 is expressed in neural populations present in adult brain areas mainly involved in locomotor and cognitive functions. The behavioral assessments have allowed us to reveal PTPH1 functionality especially within cognitive domains. Better understanding the interplay between various phosphatases regulating CNS functions, which now includes PTPH1, will be key in the future to unravel some of the complexity of CNS signaling pathways necessary for information processing.

## List of abbreviations

PTPH1: protein tyrosine phosphatase H1; KO: knock-out; WT: wild type; CNS: central nervous system; PTKs: protein tyrosine kinases; PTPs: protein tyrosine phosphatases; RPTPs: receptor-like protein tyrosine phosphatases; NRTPTs: nonreceptor PTPs; FERM: 4.1, Ezrin, Radixin, Moesin; TACE: TNFα converting enzyme;GH: growth hormone; GHR: growth hormone receptor; IGF1: insulin-like growth factor 1; BAC: bacterial artificial chromosome; ip: intraperitoneal; PBS: phosphate buffered saline; PFA: paraformaldehyde; NBT: nitrobluetetrazolium; beta-gal/LacZ: beta-galactosidase; NeuN: Neuronal Nuclei; HBSS: Hank's balanced salts solution; H2A: Histone 2A; EPM: Elevated plus maze; AUC: area under the curve; Es: embryonic stage; E2: estradiol; CA: Cornu Ammonis; DG: Dentate Gyrus; MDL: mediodorsal lateral thalamic nuclei; AV: anteroventral thalamic nuclei; VM: ventromedial thalamic nuclei; VPM: ventral posteromedial thalamic nuclei; VL: ventrolateral thalamic nuclei; VPL: ventral posterolateral thalamic nuclei; LD: laterodorsal thalamic nuclei; Po: posterior thalamic nuclei; Rt: reticular thalamic nuclei; VPPC: ventral posteromedial parvicel thalamic nuclei; DLG: lateral geniculate nucleus; PF: parafascicular thalamic nuclei; nu: nuclei; SHi: septohippocampal muclei; Tt: tenia tecta; Ig: indusium griseum.

## Competing interests

The present work is part of CP’s PhD program at the University of Eastern Piedmont, in close collaboration with MerckSerono International S.A.. MCM, PT, VM, BG, PFZ are employed by MerckSerono International S.A., which is  involved in the discovery and the commercialization of therapeutics for the prevention and treatment of human diseases.

## Authors' contributions

The study was devised by CP and MCM and carried out by CP. PT was responsible for the genotyping of all the adult animals that have been used in this study. MA performed the LacZ staining experiment on adult mice. VM and BG have been deeply involved in the first editing of the manuscript and all the authors contributed to modifications in subsequent drafts. PFZ has been involved in critically revising the manuscript and has given the final approval of the version to be published. All the authors read and approved the final version of the manuscript.

## Supplementary Material

Additional file 2 Semiquantitative RT-PCR for beta galactosidase gene in blood samples. ***a***: white and red blood cells count in PTPH1-WT and KO mice; no major differences were found in the hematological composition in WT and KO mice. ***b***: Beta-gal mRNA signal was present in PTPH1-KO hippocampus and cortex and not in the WTs; histone H2A gene was used as positive control. ***c***: RT-PCR for beta-gal/H2A on 4 increasing amounts of whole blood (WB): 5, 10, 15 and 20 μl. No signal for beta-gal or H2A was detectable using 5 and 10 μl of WB, due to the low amount of total RNA; a faint signal for H2A was detectable on 15 and 20 μl of WB and a faint band for beta-gal was present only in KO mice, representing the maximum blood contamination in the whole mouse brain. Thus, blood contamination is minimum and it cannot interfere with the main source of signal. Click here for file

Additional file 3 Additional methods. This document provides the methods and the references that have been used to perform the experiments represented in Additional file 2 Click here for file

Additional file 1 LacZ staining on PTPH1-WT and KO embryos. PTPH1-WT embryos do not show any staining either at embryological stage 14 (Es14) or at Es16. PTPH1-KO embryos display a positive LacZ staining in the hypothalamic area and but also in the dorsal root ganglia of the spinal cord, excluding the spinal cord itself. Click here for file
