# Supplementary material for: Knockout mice reveal a role for protein tyrosine phosphatase H1 in cognition
Source: Behav Brain Funct. 2008 Aug 12;4:36. doi: 10.1186/1744-9081-4-36 (PMC2531118; doi:10.1186/1744-9081-4-36)
Supplement: Additional file 2 — Semiquantitative RT-PCR for beta galactosidase gene in blood samples. a: white and red blood cells count in PTPH1-WT and KO mice; no major differences were found in the hematological composition in WT and KO mice. b: Beta-gal mRNA signal was present in PTPH1-KO hippocampus and cortex and not in the WTs; histone H2A gene was used as positive control. c: RT-PCR for beta-gal/H2A on 4 increasing amounts of whole blood (WB): 5, 10, 15 and 20 μl. No signal for beta-gal or H2A was detectable using 5 and 10 μl of WB, due to the low amount of total RNA; a faint signal for H2A was detectable on 15 and 20 μl of WB and a faint band for beta-gal was present only in KO mice, representing the maximum blood contamination in the whole mouse brain. Thus, blood contamination is minimum and it cannot interfere with the main source of signal. [file 1744-9081-4-36-S2.ppt]

## Slide 1
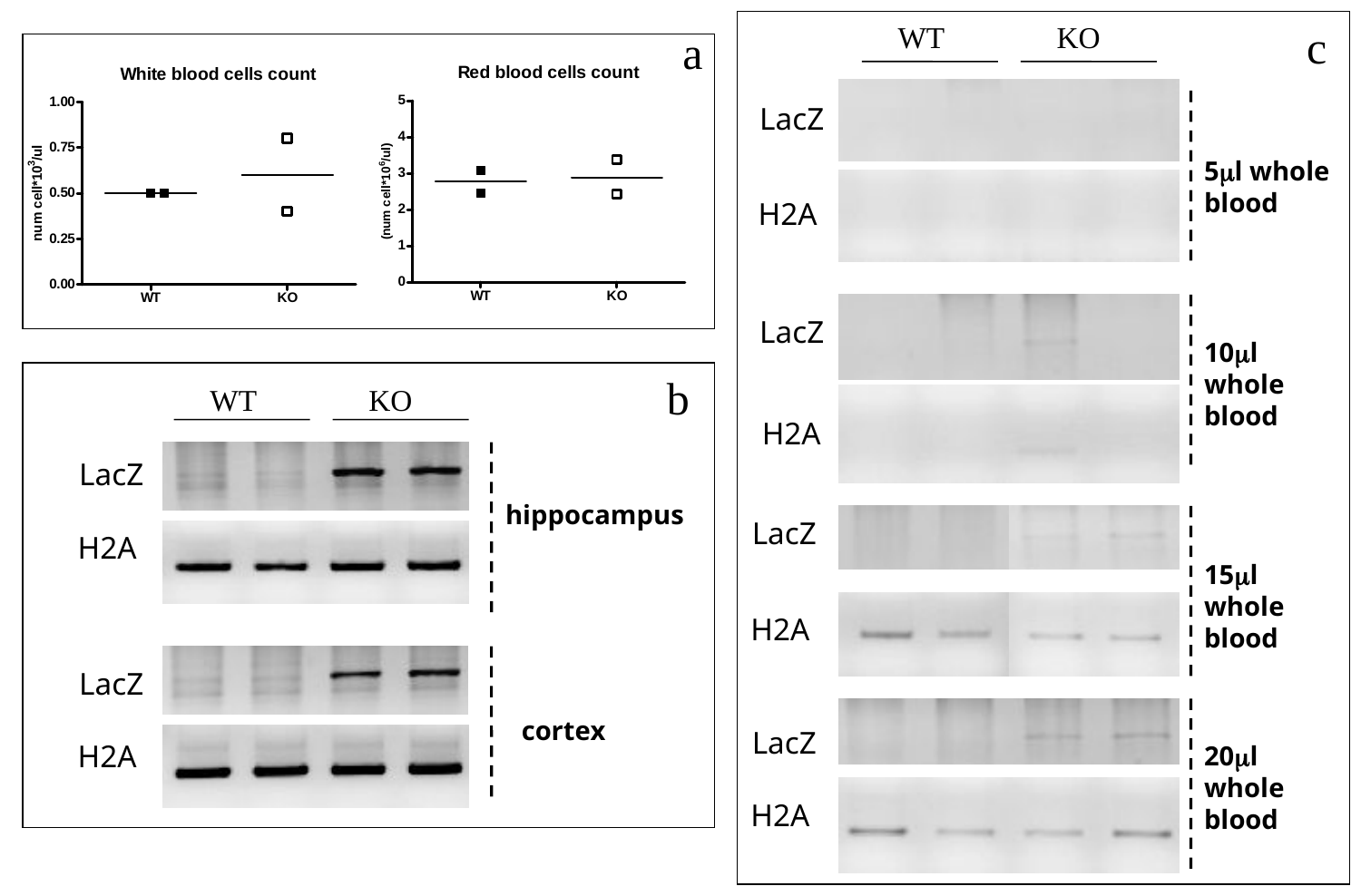

WT
KO
c
LacZ
5μl whole blood
H2A
LacZ
10μl whole blood
H2A
LacZ
15μl whole blood
H2A
LacZ
20μl whole blood
H2A
a
b
WT
KO
LacZ
hippocampus
H2A
LacZ
cortex
H2A
