# Supplementary material for: Knockout mice reveal a role for protein tyrosine phosphatase H1 in cognition
Source: Behav Brain Funct. 2008 Aug 12;4:36. doi: 10.1186/1744-9081-4-36 (PMC2531118; doi:10.1186/1744-9081-4-36)
Supplement: Additional file 3 — Additional methods. This document provides the methods and the references that have been used to perform the experiments represented in Additional file 2 [file 1744-9081-4-36-S3.doc]

# Additional methods

***Semiquantitative RT-PCR for beta galactosidase gene in blood samples***

Brains from KO and WT mice (n=2, 6 months old) were freshly removed and rinsed in HBSS. Hippocampus and cortex were dissected. Total RNA was extracted using Trizol Reagent (Invitrogen) and cleaned-up by RNAeasy columns from Qiagen. Blood was collected from the heart and was aliquoted into 5, 10, 15 and 20 μl. Total RNA from blood samples was extracted using Trizol Reagent (Invitrogen). For cortex and hippocampus 5μg of total RNA were used to perform the RT-PCR reaction (SuperScript II RT kit, Invitrogen). For 5, 10, 15 and 20 μl of whole blood, different amounts of total RNA were used for the retro-trascription (100ng, 300ng, 750ng and 1μg respectively).

## Blood cells count

The hematological analysis was carried out on 20 μl of whole blood from WT and KO mice (n=2, 6 months old) using a Coulter®Ac.T^TM^ 5diff OV (Beckam Coulter).

## Reference List

1. Koshibu K, Levitt P, Ahrens ET: **Sex-specific, postpuberty changes in mouse brain structures revealed by three-dimensional magnetic resonance microscopy.** *Neuroimage* 2004, **22:** 1636-1645.

2. Leung HW: **Pharmacokinetics and metabolism of ethylenediamine in the swiss webster mouse following oral or intravenous dosing.** *Toxicol Lett* 2000, **117:** 107-114.

3. Richter JA, Goldstein A: **Tolerance to opioid narcotics, II. Cellular tolerance to levorphanol in mouse brain.** *Proc Natl Acad Sci U S A* 1970, **66:** 944-951.

4. Swanson LW: **Mapping the human brain: past, present, and future.** *Trends Neurosci* 1995, **18:** 471-474.
