# Supplementary figures and images for: Knockout mice reveal a role for protein tyrosine phosphatase H1 in cognition
Source: Behav Brain Funct. 2008 Aug 12;4:36. doi: 10.1186/1744-9081-4-36 (PMC2531118; doi:10.1186/1744-9081-4-36)

## Slide 1
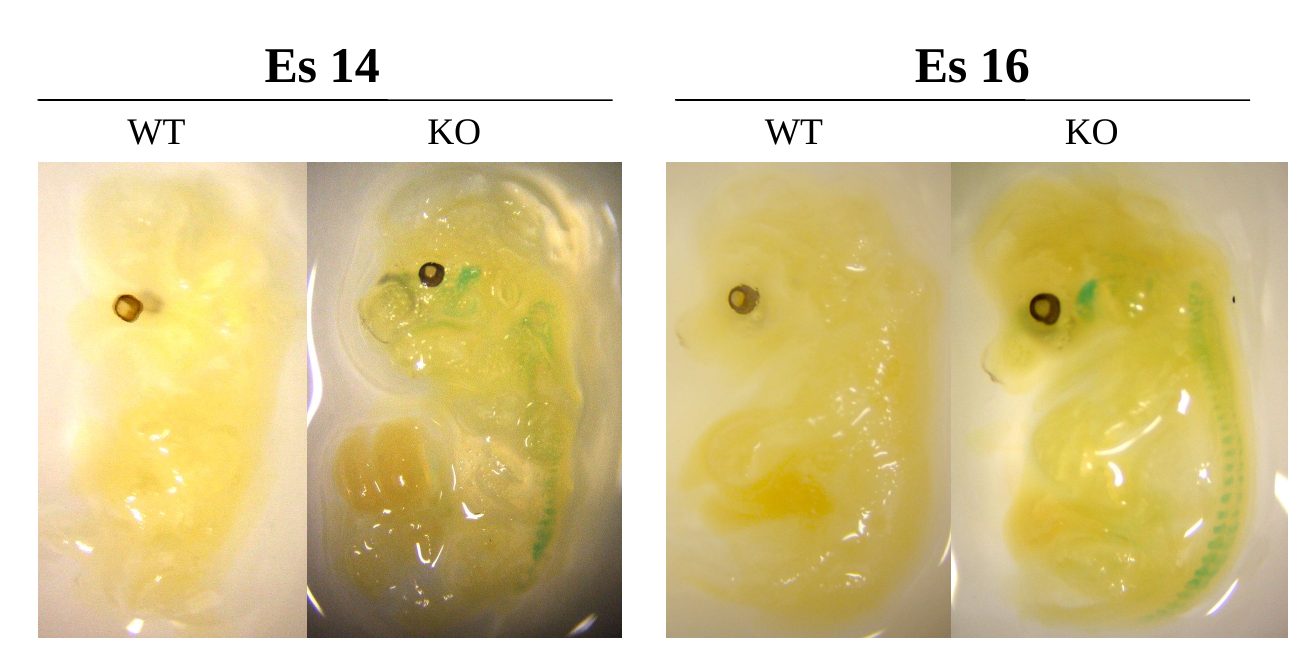

Es 14
Es 16
WT
KO
WT
KO

Supplement: Additional file 1 — LacZ staining on PTPH1-WT and KO embryos. PTPH1-WT embryos do not show any staining either at embryological stage 14 (Es14) or at Es16. PTPH1-KO embryos display a positive LacZ staining in the hypothalamic area and but also in the dorsal root ganglia of the spinal cord, excluding the spinal cord itself. [file 1744-9081-4-36-S1.ppt]
